# Metabolic rates of giant pandas inform conservation strategies

**DOI:** 10.1038/srep27248

**Published:** 2016-06-06

**Authors:** Yuxiang Fei, Rong Hou, James R. Spotila, Frank V. Paladino, Dunwu Qi, Zhihe Zhang

**Affiliations:** 1 Department of Biodiversity, Earth and Environmental Science, Drexel University, 3145 Chestnut St, Philadelphia, PA 19104.; 2 Sichuan Key Laboratory of Conservation Biology for Endangered Wildlife, Chengdu Research Base of Giant Panda Breeding, 1375 Panda Rd, Northern Suburb, FuTou Shan, Chengdu, Sichuan Province 610081, People’s Republic of China; 3 Department of Biology, Indiana Purdue University at Fort Wayne, 2101 E. Coliseum Blvd, Fort Wayne, IN 46805.

## Abstract

The giant panda is an icon of conservation and survived a large-scale bamboo die off in the 1980s in China. Captive breeding programs have produced a large population in zoos and efforts continue to reintroduce those animals into the wild. However, we lack sufficient knowledge of their physiological ecology to determine requirements for survival now and in the face of climate change. We measured resting and active metabolic rates of giant pandas in order to determine if current bamboo resources were sufficient for adding additional animals to populations in natural reserves. Resting metabolic rates were somewhat below average for a panda sized mammal and active metabolic rates were in the normal range. Pandas do not have exceptionally low metabolic rates. Nevertheless, there is enough bamboo in natural reserves to support both natural populations and large numbers of reintroduced pandas. Bamboo will not be the limiting factor in successful reintroduction.

The giant panda (*Ailuropoda melanoleuca*) is an important symbol of conservation around the world. It helps people understand and support the need for biodiversity and habitat protection. There are extensive efforts underway to increase its populations, including captive breeding, studies in nature reserves^1^,^2^ and programs for reintroduction of captive bred animals into the wild. However, we lack the basic understanding of the physiological ecology of the giant panda that is necessary to ensure that there are sufficient resources in nature reserves for reintroduced and native pandas to coexist and to adapt to the effects of climate change^3^. If we determine the metabolic cost of activity of the giant panda under natural conditions and measure the amount of food (bamboo) available in its natural environment then we can calculate upper limits on how many animals can live in a given area. Wild giant pandas eat 13.1 kg to 14.6 kg of bamboo leaves and stems a day or 43.7 kg of shoots a day^4^. Energy intake and output should be in a dynamic balance. However, differences between estimates of energy intake in food and activity metabolic rate in humans occur due to inaccurate methods^5^. Similarly, lack of validation can result in large errors in estimates of energy expenditure in animals^6^. It is essential that we have accurate data to test hypotheses about giant panda biology and to develop conservation strategies.

The giant panda is a member of the Order Carnivora, Family Ursidae, and is related to omnivorous bears^7^,^8^,^9^,^10^. It is primarily a herbivore, eating almost exclusively bamboo, although it will eat other plants and sometimes meat if available. We might expect that its metabolic rate would be below that of a generalized mammal of its size, similar to some other herbivores (e.g., sloths (*Bradypus griseus, Choloepus hoffmanni*) that feed extensively on leaves^11^. The metabolic rates of bears would give insights into the metabolic rates of giant pandas, but there are few studies of metabolism in bears and most of them are on hibernating animals^12^,^13^,^14^. In one of the few studies on bears the metabolic rate of the sloth bear (*Melurssu ursinus*) is below that expected for a mammal of its size^15^,^16^. The best way to accurately estimate the metabolic cost of activity of the giant panda is to actually measure it in the field. A recent study^17^, using the doubly labeled water (DLW) method^18^, determined that the daily energy expenditure of giant pandas was only 37.7% of the predicted values based on their body size. Here we report the metabolic rates of giant pandas measured with flow through respirometry in the laboratory and DLW in outdoor zoo enclosures containing trees, grass, wood platforms, water pools and rocks. Resting metabolic rates of giant pandas were somewhat lower than for a mammal of their size but were not exceptionally low. Activity metabolic rates were lower than those of some similar sized mammals, but those rates were measured in free-ranging individuals. Our sleeping pandas in the laboratory had higher metabolic rates than the active metabolic rates (DLW) previously measured^17^. That calls into question the previous estimates for metabolic rates for active giant pandas in the wild (Supplemental Information). Our estimated daily energy expenditure for giant pandas (DLW) was similar to estimations of daily energy intake in the wild and higher than in the previous study. Based on our data and other measurements the bamboo supply in nature reserves is not the limiting factor for giant panda populations and reintroduction programs.

## Results and Discussion

### Resting metabolic rate

We sought to measure the metabolic rate of giant pandas using DLW so that we could determine the metabolic cost of activity of free-living animals. As a first step we measured the metabolism of nine animals at rest in the laboratory at two temperatures using a Sable Systems International Flowkit- 500 mass flow system. The giant pandas were quiescent, generally asleep during the experiments, and showed no signs of stress. Then we measured the activity metabolic rates of seven animals in summer and winter at the Chengdu Research Base of Giant Panda Breeding in Chengdu China (see Methods for a complete description of the experiments).

**Figure 1:**
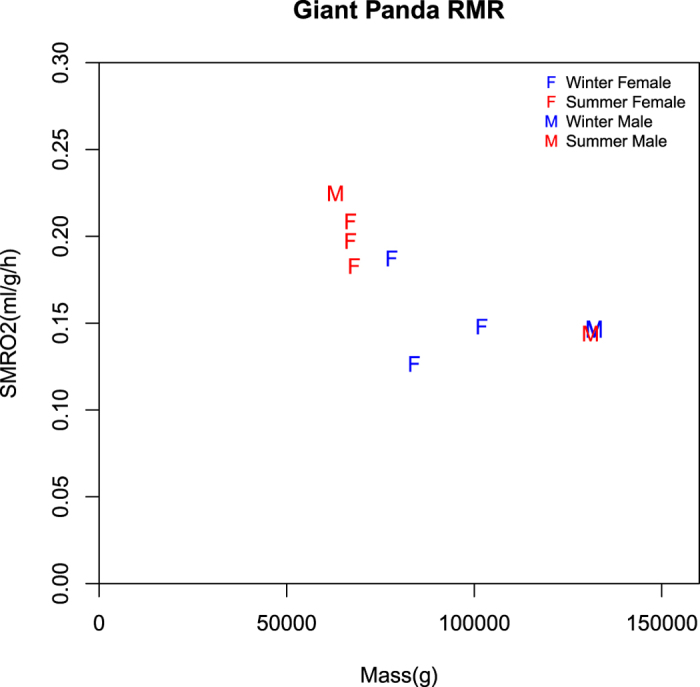
Resting metabolic rates of giant pandas measured at the Chengdu Research Base of Giant Panda Breeding in Chengdu, China. Animals were at rest in a metabolic chamber at temperatures between 9.1 and 26.5 °C. Temperatures for each experiment are provided in Table 1. M represents males and F represents females. W represents winter and S represents summer.

The resting metabolic rate (RMR) of the giant panda ranged from 0.126 ml/g/h of O_2_ to 0.225 ml/g/h of O_2_ (Fig. 1). We fit a linear model (LM) using the statistical software R to compare RMR as the response variable, to the explanatory factors of age, mass, sex, environmental temperature and season (see Methods). The LM indicated that there was a statistically significant effect of age (adult, sub-adult and young) (df = 2, 9; F = 80.16; P = 0.002), mass (df = 1, 9; F = 17.22; P = 0.025) and an interaction between sex and age (df = 1, 9; F = 37.29; P = 0.009). The mean RMR of adult and sub-adult giant pandas was 0.150 ml/g/h (n = 6, range = 0.126 ml/g/h to 0.187 ml/g/h) and for young giant pandas (1 to 2 years of age) was 0.204 ml/g/h (n = 4, range = 0.183 ml/g/h to 0.225 ml/g/h). Sex as a lone predictor was not an important influence. There was no difference in RMR between males and females, and no difference in RMR due to environmental temperature and season. The effect of mass and age were obviously related. Age affects RMR because young animals have higher RMR than adults^19^, but the statistical significance of the effects suggested that age had a greater effect than mass. Because both sub-adult pandas were female the effect of sex was not significant. Interaction of age and sex may have been related to the small sample size. These RMRs for sleeping giant pandas convert to a daily energy expenditure of 7.4 MJ/day, which is higher than the 5.2 MJ/day recently reported for active captive and wild giant pandas^17^. This difference is discussed below and in Supplemental Information.

There was no difference in metabolic rate between male and female adult giant pandas (Table 1, Fig. 1). Some mammals have behavioral and physiological differences between males and females that cause differences in RMR^20^,^21^,^22^. For example, in humans, males have higher basal metabolic rate (BMR) and active metabolic rate than females, but female margays (*Leopardus wiedii*) have higher BMR than males. Captive and wild male giant pandas are more active in the daytime than female giant pandas^23^. Female giant pandas have more restrictive habitat requirements than males and more limited home ranges^24^. Those differences could be reflected in their active metabolic rates. There was no difference in activity of males and females in our metabolic chamber. Both sexes were quiescent and usually sleeping.

Environmental temperature is an important factor affecting RMR. Mammals have a thermal neutral zone in which animals have a minimum RMR. Below that zone metabolic rate increases due to physiological heat production that maintains constant body temperature. Above that zone metabolic rate increases due to a loss in the ability of the animal to cool its body temperature by behavioral and physiological means^25^. In our experiment, there was no difference in metabolic rates of giant pandas at environmental temperatures between 9.1 °C and 26.5 °C. Therefore, those temperatures were within the thermal neutral zone. There was no indication that the animals were more active at these temperatures and they showed no signs of behavioral stress.

The respiratory quotients (RQ) that reflect CO_2_ production divided by O_2_ consumption were variable. Values ranged from 0.59 to 0.85 (Table 1). An animal oxidizing fat has a RQ of 0.7, an animal oxidizing carbohydrates has an RQ of 1.0 and an animal oxidizing protein has an RQ of 0.8–0.9. RQ values lower than 0.7 are often considered to be in error^26^. However, low RQs are not unusual in metabolic studies of animals. For example, studies on birds^27^, hibernating black bear (*Ursus americanus*)^28^, pig (*Sus scrofa*)^29^,^30^, white rat (*Rattus norvegicus*)^31^, and green turtle (*Chelonia mydas*)^32^ report some RQs below 0.7. Low RQs also occur in humans due to fatty acid desaturation^33^. Low RQs may be due to fat transformation into carbohydrate through the process of gluconeogenesis^34^,^35^, incomplete oxidation of fat and non-pulmonary CO_2_ loss^36^, incomplete ketone oxidation and the loss of oxidation products through urine or breath^37^. Elevated RQs above 0.7 were probably due to animals that were not post-absorptive^26^,^36^. Giant pandas only fasted for 12 h because if they fasted longer they became restless and active.

Our understanding of the physiological patterns and biochemical processes reflected in these differences in RQ is rudimentary. Differences in metabolism in respiratory studies are magnified by factors such as phylogeny, nutritional status and history, tissue synthesis and energetic challenges. However, the accuracy of flow through respirometry is within 0.4% when instruments are calibrated against standard test gases and a known oxidative process^6^. Therefore, our laboratory measurements provide the best available estimate of metabolism in giant pandas. The exceptional low daily energy expenditures reported for active giant pandas^17^ are not consistent with our values for resting animals.

Resting metabolic rate of the giant panda was somewhat lower than that of other mammals of the same size (Table 2). The metabolic rate of the giant panda was higher than that of the sloth bear but lower than that of the tiger (*Panthera tigris*), lion (*Panthera leo*), cow (*Bos taurus*), and eland (*Taurotragus oryx*). At similar mass, humans have a RMR similar to that of young (2 year old) giant pandas. Dolphins and sea lions have higher metabolic rates. This is because water has a much higher heat transfer rate than air and the higher rate of heat loss in water requires a higher RMR^38^.

**Figure 2:**
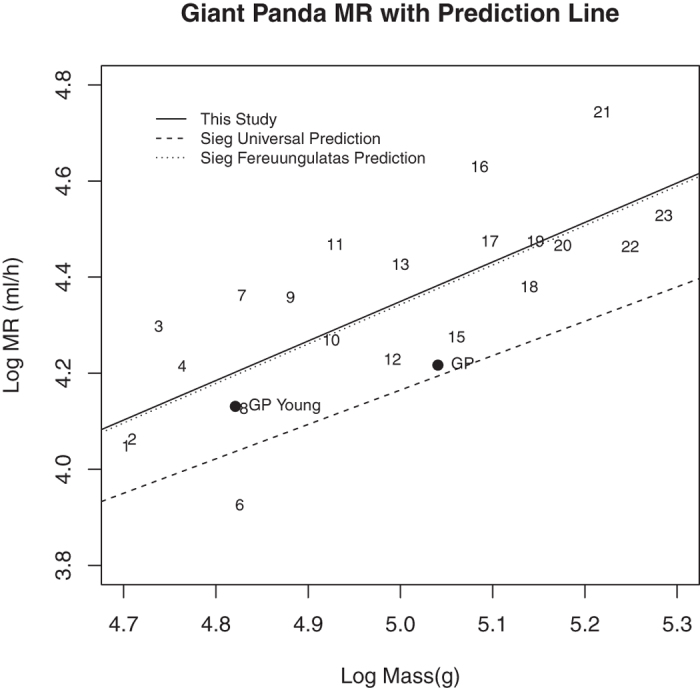
Relationship between body mass and resting metabolic rate in giant pandas and 21 other large mammals. GP represents giant panda. Regression lines for all mammals and for Ferreuungulate mammals are from Sieg *et al.*^16^. Solid line is regression line calculated by us with the addition of the giant panda.

We compared the resting metabolic rate in ml/h (MR) of the giant panda to those reported for 21 other mammals ranging in size from 50 kg to 193 kg taken from Sieg *et al.*^16^ (Fig. 2). The regression line through the combined data (log_10_ (MR) = 0.8227 Log_10_ (Mass) +0.2359, r^2^ = 0.57; P = 0.000) was almost the same as that of Sieg *et al.*^16^ for carnivores/ungulates/pangolins (Fereuungulata)^39^,^40^. That was not surprising since we only added two data points. Both of the regression lines were above the line calculated from all 695 mammals in Sieg *et al*’s data set. That is consistent with their conclusion that phylogenetic relationships affect the body size- metabolic rate regression and that there is not a single universal metabolic rate-body mass scaling relationship in mammals.

Giant panda metabolic rates (Table 1) were 6.0% to 44.3% below those predicted by the Fereuungulata regression line. White and Seymour^41^ suggest that the presence of large herbivores in a data set will elevate the scaling exponent in body mass to metabolic rate regressions because large herbivores are less likely to be post-absorptive when metabolic rate is measured. In support of this suggestion our data indicated that even if some of our giant pandas were not post-absorptive, their metabolic rates were still below predicted values for mammals of similar size. Therefore, a combination of phylogenetic relationships and physiological factors affect the metabolic rate of individual species and no one predictive line can account for all variation in the body size-metabolism relationship among mammals.

### Activity Metabolic Rates

We measured field metabolic rate (FMR) using DLW in seven experiments on six individuals in two different seasons (Table 3). The CO_2_ production was 0.265 ml/g/h and 0.658 ml/g/h for two individuals over 3–5 days (Mean = 0.462 ml/g/h) in winter (mean temperature = 8.6 °C, range = 5.0 °C to 18.0 °C during experiment) and 0.126 ml/g/h −0.404 ml/g/h for five individuals over 5 days (mean = 0.256 ml/g/h; SD = 0.126) in summer (mean temperature = 25.2 °C, range = 22.0 °C to 33.0 °C during experiment). The O_2_ consumption was calculated to be 0.295 ml/g/h and 0.731 ml/g/h (n = 2, mean = 0.513 ml/g/h) in winter and 0.140 ml/g/h −0.449 ml/g/h (n = 5, Mean = 0.284 ml/g/h; SD = 0.140) in summer. Total body water percentage was 63.4% to 75.7%. Water turnover rate was 15.52 kg/day (SD = 4.44) or 17.45% of total body water/day (SD = 3.15%). Isotope half-life was 2.28 days to 3.81 days. There was considerable individual variation in these values (Table 4).

The water-loop of the giant panda was much faster than predicted. Speakman^18^ predicted that a 50 kg or larger animal would have a 5-day half-life for doubly labeled water. However, a 100 kg giant panda had a rate twice as high as the prediction. This was probably due to their special diet, 99% bamboo, which had a high water content. Giant pandas did not drink water very often, but they did appear to drink a lot during each episode. We observed them drinking for 2 to 3 min at a time and estimated that they took up a liter or more each time. In addition, the giant panda has a different kidney type from other bears^8^. The kidney of the giant panda is composed of 6 to 11 renal lobes. Each renal lobe is comprised of 2–3 primary small kidneys, which is an archaic type. The kidney of other ursids is duplex, built up by many individual renculi^42^.

The FMR of the giant panda was lower than those of some similar sizes mammals^43^ (Table 5), including the seal (*Arctocephalus gazella*), deer (*Odocoileus hemionus* and *Cervus elaphus*), oryx (*Oryx leucoryx*) and kangaroo (*Macropus giganteus*). However it was higher than that of the reindeer (*Rangifer tarandus*). It is not surprising that the FMRs of our giant pandas were lower than those of similar sized mammals, since the pandas were in a captive zoo-like environment and were not very active while the other animals were free ranging. The low FMR of the reindeer may have been because they were in an energy conserving mode due to cold conditions in the field.

The active metabolic rates of giant pandas varied between individuals despite the active time for each individual being very similar. The daily timetable was very regular for each giant panda, no matter the season. They usually were active from 0730 to 1200. This was the most active time during the day, but they would rest periodically during that time. Then they would rest until 1600 with short periods of activity. After that they were awake, eating for about 1 or 2 h and occasionally walking about. After 1900, they would sleep until 0700. However, most giant pandas would wake up once or twice to eat some bamboo during the night.

The active time was greatly influenced by husbandry practices. Keepers usually cleaned the large cage of a giant panda inside a building and gave the animal new bamboo at 0730. At that time the giant panda was allowed out into the enclosure. However, if the temperature was greater than 25 °C in the summer, keepers would keep giant pandas inside the building so that they did not get heat stressed. Husbandry experience indicated that giant pandas would have health problems after long exposure to temperatures higher than 25°C. The keepers would add new bamboo in the afternoon around 1600 and call the giant panda back to the cage. Giant pandas were less active in the summer than in the winter. In the summer, giant pandas rarely walked around. They just ate and drank water, or found a cool spot to rest. In winter, they actively moved around in the enclosure. They climbed trees, played with each other, made scent marks on trees and other objects and even watched people who were watching them. We recorded their active time but were unable to measure the magnitude of activity. That explains why they had similar activity times but quite different FMRs. Giant pandas were active about 40.0% of the time during the winter DLW experiments and 30.3% to 34.0% in the summer experiments. There was no direct relationship between the activity times of the giant pandas and their active metabolic rates. The difference in FMR in summer and winter was illustrated by looking at the same individual. Panda #467 had a much higher active metabolic rate in winter than in summer.

We used 0.9 as the RQ to convert oxygen consumption to kilojoules because the animals were eating bamboo supplemented with foods such as apples and “panda cake”, a biscuit made of a mixture of grains with vitamins. The diet was mostly carbohydrate with some protein. The RQ for carbohydrates is typically 1.0 and that for proteins is about 0.8–0.9. The values of RQ in the laboratory study were lower because the animals did not eat for 12 hours. The average daily energy expenditure (DEE) was 21,592 KJ/day (SD = 13,323, range = 9,401 KJ/day to 47,716 KJ/day). These values were about three times higher than the resting metabolic rate in our study. They were four times higher than the activity metabolic rate for captive animals and 3.5 times the rate for wild animals in the previous study by Nie *et al.*^17^. The differences in activity metabolic rates between the two studies are surprising since our giant pandas were relatively inactive in a zoo like setting and the animals in the previous study were apparently more active, especially in the wild. With greater activity levels, their metabolic rates should have been even higher than those that we measured.

It is difficult to determine the reasons for these differences in estimates of metabolic rates in the two studies. Our resting metabolic data were obtained using standard procedures for flow through respirometry using Sable Systems instruments (see Methods). Our activity metabolic rates were consistent with what was expected for active vs resting mammals. That is, active metabolic rates were about three times resting metabolic rates^25^,^43^. We cannot determine the causes for the very low activity metabolic rates reported in the study by Nie *et al.*^17^ based on the methodological information available in that paper. It is possible that their pandas did have exceptionally low metabolic rates, but those low metabolic rates may have been induced by their methods or the animals may have been particularly lethargic (see Supplementary Information for further discussion). Only further studies using DLW in giant pandas under natural conditions will clarify the differences between our two studies.

### Ecological Implications

The mean DEE of 21,592 KJ per day in our study was similar to the estimation of daily digestible energy intake (17,222 KJ to 28,329 KJ) of wild giant pandas^4^. Giant pandas would have to eat about 13.1 kg of bamboo to support the active metabolic rates that we measured^2^,^4^. Giant pandas at the Panda Base usually ate around 15 kg to 20 kg bamboo per day. Giant pandas in the Xiangling Mountains eat 13.1 kg to 14.5 kg of bamboo a day^4^.

This suggests that bamboo is not a limiting factor in the number of giant pandas that can live in a given nature reserve. Any extrapolation is of course over simplistic. However, we can do some first order calculations to get an overview of the situation. For example, in the Yele Nature Reserve, there are 1,634,529.3 kg of one species of bamboo alone (*Bashania spanostachya*)^2^ per km^2^. Based on our FMR measurements and digestive efficiency measurements^4^ a giant panda needs to eat about 20 kg of bamboo a day. That means there is enough bamboo in 1 km^2^ of the Reserve to provide food for 81,726 panda days. Assuming that the giant pandas eat no more than ½ of the standing crop of bamboo then that would provide about 40,000 food days, sufficient for up to 110 pandas in a year. If they used only 10% of the bamboo resources a year, then 1 km^2^ would support 22 giant pandas, and that is for just one species of bamboo in that reserve. However, the home range of a giant panda^44^,^45^ is usually 3.0 to 6.0 km^2^. There must be limitations in the biology of the giant panda that go beyond the bamboo supply, since that size home range provides a density of giant pandas that is 1 to 2% of what would be supported by bamboo alone.

In another example, based on food supply the 2000 km^2^ Wolong Nature Reserve, with 50% undisturbed area^46^,^47^ could support 22,000 giant pandas if all of the area is covered with bamboo, but only 166 to 333 giant pandas based on home range. The estimated number of giant pandas living in the reserve is 143 as reported by the State Forestry Administration of the People’ Republic of China in 2003. If the disturbed area was rehabilitated as giant panda habitat the number of giant pandas in the Reserve could be doubled because the amount of bamboo would be doubled and the habitat available for pandas would be doubled. Therefore, bamboo supply is not the limiting factor for natural giant panda populations or for reintroduction of giant pandas into reserves. The key limiting factors are anthropogenic disturbance and perhaps behavioral interactions between animals living there. Of course all of this can change if climate change causes the elimination of large areas of bamboo in these and other nature reserves as predicted for the Qinling Mountains^3^.

## Methods

### Giant panda acquisition and maintenance

We studied giant pandas (*Ailuropoda melanoleuca*) at the Chengdu Research Base of Giant Panda Breeding (Panda Base) (www.panda.org.cn) and conducted all experiments in cooperation with the research, veterinary and husbandry staffs there. The Chengdu Research Base of Giant Panda Breeding is a nonprofit organization with offices in Chengdu, Sichuan Province, China. It was a center for wildlife research, giant panda captive breeding, conservation education, and educational tourism. It was difficult to carry out metabolic studies of giant pandas in the past because they were very rare and most zoos only had one or two individuals. The Panda Base had 107 giant pandas and allowed us to use nine of them for the laboratory experiments and eight of them for the DLW experiments under very close veterinary supervision. Giant pandas lived in enclosures singly or in small groups and ate a diet composed primarily of bamboo supplemented with foods such as apples and “panda cake”, a biscuit made of a mixture of grains with vitamins. The enclosures were about 500 m^2^ with trees, grass, wooded platforms, pools of water and rocks. Giant pandas had various objects such as tires and large balls as “toys” in the enclosures for behavioral enrichment. Animals lived in the enclosures year round and were brought into a building at night and if air temperature rose above 25 °C. We transported pandas to the laboratory for each resting metabolic rate experiment. During the DLW experiments giant pandas remained in their normal enclosures.

This study was approved by the Chengdu Research Base of Giant Panda Breeding and the Institutional Animal Care and Use Committee of Drexel University (Protocol #20032). The methods used were in accordance with the approved guidelines of these institutions and followed all regulations of the Research Base and Drexel University. Permission to work at the Panda Base was given by the Director after consultation with the Research, Husbandry and Veterinary Departments. No animals were sacrificed during the experiments. No anesthesia was used. The research protocol, capture methods, and handling procedures were approved by the Directors and staff of the Research Department, Veterinary Department, and Husbandry Department of the Chengdu Research Base of Giant Panda Breeding. There was no animal care and use committee at the Panda Base. Instead the Research Department, Veterinary Department, and Husbandry Department reviewed the protocol and determined that it was safe for the animals being studied. Approval came from each department. Then the overall approval came from the Director. Giant Pandas were kept in enclosures at the Panda Base and were free to move about their enclosures. Animals were called into transport cages, handled and moved to the laboratory by the husbandry staff and experiments were conducted under veterinary supervision. For the DLW experiments, animals were trained to present their forearm for blood sampling making it possible to obtain samples with minimum disturbance to the animal. We weighed animals on a scale to +/− 0.05 kg.

### Resting metabolic rate experiment

We measured resting metabolic rate during two seasons, summer and winter. Because there was no effective air temperature-control room at the Panda Base we had to use natural air temperature change during the seasons to study pandas under warm and cool conditions. We did that to assess the thermal neutral zone of the giant panda. However, according to the husbandry rules of the Panda Base, giant pandas should not be exposed to temperatures greater than 25 °C. Past experience showed that if giant pandas experienced temperatures above 25 °C, they became heat stressed and experienced health problems. Therefore, in our experiment, we attempted to keep the maximum experimental temperature at 25.0 °C. In winter we could not obtain an experimental temperature below 9.1 °C.

We studied five giant pandas during each season, including young animals (1–2 years old), sub adults and adults. One adult was studied twice. Because giant pandas are diurnal, we conducted all experiments during night hours (2200–0400). Giant pandas were weighed before and after each experiment. We recorded data every 20 minutes during the experiment and reported the lowest values recorded for each experiment in Table 1.

Our goal was to measure the basal metabolic rate (BMR) of these animals keeping in mind the criteria of Kleiber^48^ that the animals be post-absorptive and at rest. Normally we would fast the animals for 24 h before an experiment. However, past experience at the Panda Base indicated that if giant pandas did not eat for 24 h they became restless and agitated, paced around their enclosures and were very active. Therefore, animals fasted for 12 h before an experiment, but could drink water. Some animals did pass feces during experiments so they may have been digesting vegetation. Speakman *et al.*^49^ stated that it is not always possible to adhere completely to the Kleiber criteria in studies on wild animals and that it is necessary to trade off the strict adherence to arbitrary rules with the constraints of reality for the species under study. Even Kleiber^48^ stated that measurement of a true BMR was probably only possible in humans. Many authors use the term standard metabolic rate or resting metabolic rate rather than BMR for non-human animals. We believe that our measurements of the resting metabolic rate (RMR) of giant pandas are as close to BMR as it is possible to obtain under realistic conditions because the animals were asleep in the experimental chamber during most of the experiment. We used data from those periods when the animals were asleep (lowest values) and did not use data from any periods when the animals were active.

We measured metabolic rate in a Plexiglas chamber using a flow through system to measure oxygen consumption and carbon dioxide production. The chamber was 1.5 m * 1.5 m * 2.0 m and constructed of 2.0 cm Plexiglas with a steel frame for added strength. One side of the chamber was a door held by steel hinges, sealed with a rubber gasket and closed with metal latches. There were three 2.5 cm holes with 60 cm long tubing attached to avoid backflow for air intake at the bottom right side of the chamber. There was one 2.5 cm exit hole at the top left side of the chamber that connected to spiral-wound tubing leading to a Flowkit -500 mass flow system (Sable Systems International). A subsample of air went from the Flowkit pump to a FOXBOX oxygen and carbon dioxide analyzer (Sable Systems International). The three air intake holes and one air exit hole eliminated negative pressure in the system. The placement of the holes reduced air stagnation and two small battery operated fans in the chamber assured that the air was well mixed. Six 24-gauge Cu-Co thermocouples (+/− 0.05 °C) located inside the chamber on the top, right side, left side, back side, and in the mouth of the air intake and exit holes measured chamber temperatures.

The Sable System Flowkit used a precision mass flow sensor with a rotary pump controlled by a microprocessor to control air flow rate to within 2% of reading. The Flowkit pump’s air flow was set at 150 L/min. After leaving the Flowkit pump, air was subsampled though a small plastic tube and drawn into the FOXBOX system at a rate of 200 ml/min. The subsample went through a relative humidity meter and temperature meter before it entered the gas analyzers. Sample air passed through the CO_2_ analyzer and then a drierite (anhydrous calcium sulfate (gypsum) with cobalt (II) chloride added as a color indicator) column before entering the O_2_ analyzer to remove water vapor, which would interfere with the fuel cell in the oxygen analyzer. The accuracy of the Sable System Foxbox was 0.1% for O_2_ over a range of 2–100% and 1% for CO_2_ over a range of 0–5% when calibrated used using calibration gas (14.93% O_2_, 3.99% CO_2_) from Dalian Special Gas Industry Company and tested by National Institute of Measurement and Testing Technology. We also used 100% dry N_2_ and room air to calibrate the system. The Sable Systems instrument converted gas measurements to standard temperature and pressure dry (STPD).

### DLW experiments

There was no information on equilibration time and water loop rate of giant pandas or similar animals when we did the experiments. Therefore, we picked one giant panda to do a safety test to be sure that there was no harm to the animal and also to measure the equilibration time. We took a background blood sample first, and then injected 10.12 g of doubly labeled water (Sigma-Aldrich deuterium oxide-18, 99% D, 75% O18) mixed with physiological saline. The dose depended upon mass following guidelines by Speakman^18^, such that we obtained 80 p.p.m. oxide-18 above the background level at equilibration time. After two physiological half-lives the concentration would be 20 p.p.m. above background level, which was the minimum concentration that the mass spectrometer could accurately measure. The elimination half-life is predicted to be 5 days for a 50 kg mammal or larger^18^. That meant that there would be about 10 efficacious experimental days. After injection, we took blood samples every 2 h for 8 h to measure the equilibration time. Then, we took blood samples after 3 days, 5 days and 10 days to measure the physiological half-life. We sealed all blood samples in individual glass tubes using an alcohol burner, placed them in a bigger PVC tube with cotton to protect them and stored them in a freezer at −40 °C.

For a normal experiment, we took a background blood sample first and injected 10.15 g to 12.56 g DLW depending on the mass of the panda. After 5 h equilibration time, we took a sample. After 3 days and 5 days we took additional blood samples, treating them as before. We measured the isotope background level, dilution space and isotope turnover rates for each giant panda (Table 6).

We also set up a video camera for each experimental animal to record its behavior during the experimental period in summer. The camera operated 24 h a day from the beginning of the first blood sample to the end of taking the last blood sample. We used this record to calculate the active time of the animal. The camera was not available for winter experiments.

Samples were tested and analyzed by the Laboratory of Isotope Geology at Chengdu University of Technology (formerly: Chengdu College of Geology). Laser spectroscopy (Isotopic Water Analyzer (912-0026), Los Gatos Research, USA) was used to test samples. Each sample was tested three times (average standard error was 2.35%). We also used a mass spectrometer (MAT253, Thermo Finnigan, Germany) to do comparison with the laser spectroscopy. We used standard water, goat blood and giant panda blood as standard material to compare these two instruments. The measurements between the two methods had a 3.79% difference. We used the two-sample technique^18^ to calculate the CO_2_ production. We used 0.9 as RQ to predict oxygen consumption because animals were eating bamboo supplemented with foods such as apples and “panda cake”, a biscuit made of a mixture of grains with vitamins. The diet was mostly carbohydrate with some protein. The RQ for a diet of carbohydrate is 1 and the RQ for a diet of protein is about 0.8–0.9. Assuming that FMR CO_2_ is 0.658 ml/g/h, if RQ was 0.8 then FMR in O_2_ would be 0.822 ml/g/h rather than 0.731 ml/g/h from line 1 in Table 3. If RQ was 1, then FMR in O_2_ would be 0.658 ml/g/h. The FMR in O_2_ changes by about 10 to 12% depending upon what RQ is assumed. That change would not greatly affect the resulting conclusions about FMR. We used Microsoft Excel to store data and for calculations.

### Statistical analysis

We fit a linear model (LM) using the statistical software R (R Development Core Team 2011) for the resting metabolic rate experiments. The LM included the RMR of O_2_ as the response variable, and age, mass, sex, temperature and season as explanatory factors. We used Akaike information criteria (AIC) as a measure of the relative quality of the statistical models to remove factors that were not significantly related to RMR, and compared the full and reduced models using residual sum of squares (RSS) criteria. The final linear model contained the effects of mass and age. We accepted P ≤ 0.05 as a statistically significant difference. We used descriptive statistics to describe the DLW results.

## Additional Information

**How to cite this article**: Fei, Y. *et al.* Metabolic rates of giant pandas inform conservation strategies. *Sci. Rep.* **6**, 27248; doi: 10.1038/srep27248 (2016).

## Supplementary Material

Supplementary Information

## Figures and Tables

**Table 1:** Resting metabolic rates and respiratory quotients (RQs) of giant pandas measured in a metabolic chamber at the Chengdu Research Base of Giant Panda Breeding in Chengdu, China.

Studbook Number	Sex	Mass (g)	Age	Temperature (°C)	High (°C)	Low (°C)	RMR CO_2_ (ml/g/h)	RMR O_2_ (ml/g/h)	RQ
386	M	132000	A	9.1	10.6	8.4	0.121	0.146	0.83
762	F	84000	S	9.2	10.7	8.4	0.107	0.126	0.85
761	F	78000	S	9.2	10.5	8.2	0.129	0.187	0.69
467	M	132000	A	12.8	14.3	11.9	0.113	0.147	0.77
491	F	102000	A	10.8	11.4	9.9	0.089	0.148	0.60
386	M	131000	A	26.5	26.8	25.8	0.104	0.144	0.72
814	F	67000	Y	25.0	25.5	24.4	0.125	0.208	0.60
813	F	67000	Y	24.9	25.3	24.4	0.136	0.198	0.69
815	M	63000	Y	25.5	25.7	25.2	0.133	0.225	0.59
820	F	68000	Y	25.3	25.7	24.9	0.140	0.183	0.77

A is adult, S is subadult, and Y is young. M is male and F is female.

**Table 2:** Metabolic rates of 21 large mammals compiled by Sieg *et al.* 16 and juvenile and adult giant pandas measured at the Chengdu Research Base of Giant Panda Breeding.

Number	Animal	Mass (g)	RMR O_2_ (ml/g/h)	Log10 (Mass)	Log10 (MR O_2_)
1	Jaguar	50400	0.222	4.70	4.05
2	White Tailed Deer	51190	0.226	4.71	4.06
3	Ribbon Seal	54700	0.363	4.74	4.30
4	Red Deer	58000	0.283	4.76	4.22
5	Giant Panda Young*	66250	0.204	4.82	4.13
6	Sloth Bear	66957	0.126	4.83	3.93
7	Bighorn Sheep	67332	0.342	4.83	4.36
8	Homo sapiens	67650	0.198	4.83	4.13
9	American Badger	76020	0.300	4.88	4.36
10	Arabian Oryx	84100	0.221	4.92	4.27
11	Caribou	85000	0.346	4.93	4.47
12	Lion	98000	0.173	4.99	4.23
13	Water Buck	100000	0.267	5.00	4.43
14	Giant Panda Adult*	109833	0.150	5.04	4.22
15	Llama	115000	0.164	5.06	4.28
16	Sea Lion	121833	0.350	5.09	4.63
17	Eland	125000	0.239	5.10	4.48
18	Tiger	137900	0.174	5.14	4.38
19	Wildebeest	140000	0.213	5.15	4.47
20	Harp Seal	150000	0.195	5.18	4.47
21	Bottlenosed Dolphin	165625	0.335	5.22	4.74
22	Ass	177500	0.164	5.25	4.46
23	Cow	193000	0.175	5.29	4.53

*Indicates data collected in this study.

**Table 3:** Field metabolic rates (FMRs) of giant pandas measured with doubly labeled water at the Chengdu Research Base of Giant Panda Breeding in Chengdu, China.

Studbook Number	Sex	Mass (kg)	Season	Average Temperature (°C)	High (°C)	Low (°C)	Study Duration (day)	Active Time (%)	FMR CO_2_ (ml/g/h)	FMR O_2_ (ml/g/h)	FMR(KJ/day)
467	M	132	W	8.9	17.0	5.0	2.75	40.0	0.658	0.731	47717
491	F	102	W	8.3	18.0	5.0	5.50	40.0	0.265	0.295	14876
649	M	114	S	25.9	32.0	22.0	4.81	34.0	0.404	0.449	25301
467	M	136	S	25.9	32.0	22.0	4.83	32.4	0.126	0.140	9401
574	M	138	S	24.5	31.0	22.0	4.80	30.3	0.177	0.197	13450
630	M	122	S	25.0	31.0	22.0	4.80	33.9	0.193	0.215	12959
540	M	132	S	24.5	33.0	22.0	4.80	33.6	0.378	0.420	27440

W represents winter, S represents summer, M represents male and F represents female. The FMR O_2_ was calculated using an RQ of 0.9.

**Table 4:** Water turnover in giant pandas measured with doubly labeled water at the Chengdu Research Base of Giant Panda Breeding in Chengdu, China.

Studbook Number	Sex	Mass (kg)	Season	Water loss (kg/day)	Water Turnover (%/day)	Total Body Water (%)	Half Life (day)
467	M	132.0	W	16.93	16.93	75.7	2.95
491	F	102.0	W	8.89	13.13	66.4	3.81
649	M	114.0	S	12.76	15.56	72.0	3.21
467	M	136.0	S	19.22	20.53	68.8	2.44
574	M	138.0	S	21.08	21.97	69.5	2.28
630	M	122.0	S	11.77	15.20	63.4	3.29
540	M	132.0	S	17.99	18.85	72.3	2.65

W represents winter, S represents summer, M represents male and F represents female.

**Table 5:** Field metabolic rates (FMR) of large mammals from our study and other investigators^43^.

Genus	Species	Common name	Mass (kg)	SD	FMR (KJ/day/kg)	SD
*Arctocephalus*	*gazella*	fur seal	43.51	2.58	417.74	66.78
*Lama*	*glama*	camelid	48.00	6.62	297.53	57.98
*Odocoileus*	*hemionus*	mule deer	53.03	12.52	654.30	191.11
*Macropus*	*giganteus*	grey kangaroo	60.80	0.00	176.48	0.00
*Phocarctos*	*hookeri*	sea lion	61.03	18.09	264.02	64.36
*Rangifer*	*tarandus*	reindeer	74.93	4.61	106.88	55.87
*Oryx*	*leucoryx*	oryx	84.10	13.86	196.00	73.33
*Cervus*	*elaphus*	red deer	107.50	1.86	234.87	20.13
*Pongo*	*pygmaeus*	orangutan	113.91	36.10	472.90	28.84
*Ailuropoda*	*melanoleuca*	giant panda	125.14	13.21	172.85	100.69
*Odobenus*	*rosmarus*	walrus	1310.00	84.85	292.87	58.05

**Table 6:** Isotope background level, dilution space and isotope turnover rates for giant pandas in activity metabolic rate experiments at the Chengdu Research Base of Giant Panda Breeding in Chengdu, China.

Studbook Number	D2 Background (ppm)	O18 Background (ppm)	Nd (mol)	No (mol)	Nd/No	kd	ko	kd/ko
467	148.45	1988.17	5671.33	5539.46	1.0238	0.0108	0.0128	0.8429
491	147.65	1985.73	3861.97	3752.99	1.0290	0.0076	0.0086	0.8778
649	152.59	1993.14	4712.26	4544.81	1.0368	0.0090	0.0104	0.8656
467	146.13	1984.39	5597.90	5187.36	1.0791	0.0119	0.0128	0.9295
574	146.81	1983.81	5509.94	5314.54	1.0368	0.0127	0.0138	0.9223
630	148.72	1986.31	4484.69	4288.52	1.0457	0.0088	0.0097	0.9022
540	149.82	1988.67	5457.22	5287.02	1.0322	0.0109	0.0123	0.8840

The kd is mean isotope turnover rate of D2 and ko is mean isotope turnover rate of O18. Nd is the isotope dilution space of D2 and No is the isotope dilution space of O18.
