# Corrigendum: Metabolic rates of giant pandas inform conservation strategies

**DOI:** 10.1038/srep33268

**Published:** 2016-09-21

**Authors:** Yuxiang Fei, Rong Hou, James R. Spotila, Frank V. Paladino, Dunwu Qi, Zhihe Zhang

Scientific Reports 6: Article number: 2724810.1038/srep27248; published online: 06 06 2016; updated: 09 21 2016

While this paper was under review, a correction was published to reference 17, which indicated that the reported dosage rate was incorrect. Therefore, points 2–4 in the Supplementary Information of this paper cannot explain the reported discrepancy in metabolic rates. The authors apologize for this oversight.

