# Supplementary material for: Metabolic rates of giant pandas inform conservation strategies
Source: Sci Rep. 2016 Jun 6;6:27248. doi: 10.1038/srep27248 (PMC4893702; doi:10.1038/srep27248)
Supplement: Supplementary Information [file srep27248-s1.doc]

**Supplementary information**

**Metabolic rates of giant pandas inform conservation strategies**

Yuxiang Fei^1^, Rong Hou^2^, James R. Spotila^1*^, Frank V. Paladino^3^, Dunwu Qi 2’

and Zhihe Zhang^2*^

^1^Department of Biodiversity, Earth and Environmental Science, Drexel University,

3145 Chestnut St, Philadelphia, PA 19104

^2^ Sichuan Key Laboratory of Conservation Biology for Endangered Wildlife, Chengdu Research Base of Giant Panda Breeding, 1375 Panda Rd, Northern Suburb, FuTou Shan, Chengdu, Sichuan Province 610081, People’s Republic of China

^3^Department of Biology, Indiana Purdue University at Fort Wayne, 2101 E. Coliseum Blvd, Fort Wayne, IN 46805

*Corresponding authors

E-mail: [spotiljr@drexel.edu](mailto:spotiljr@drexel.edu)

**Comments on Metabolic rates reported by Nie et al 2015 Science 349:171-174**

The active metabolic rates reported by Nie et al^17^ were much lower than the resting metabolic rates measured in our study using flow through respirometry. The giant pandas in our study were generally asleep. It does not seem possible that the active metabolic rates could be lower than resting metabolic rates. There are two possibilities to explain this discrepancy. First the giant pandas in the Nie study could have been abnormally inactive or even sub-clinically ill. Second, there may be important differences in methodology between our studies. We list 5 such considerations below:

1. Nie et al used 0.8 as the RQ (respiratory quotient) to calculate energy consumption. Giant pandas consume primarily carbohydrates, so using 0.9 would be a better option. This would cause only a minor difference (about 10 %) in the active metabolic rate estimate. If we used 0.8 it would have resulted in a higher estimate for oxygen consumption (Table 3).
2. Nie et al injected 0.3 g DLW per 100 g body mass. If a giant panda weighed 100 kg, they injected 300g DLW. They used 639,700 ppm ^18^O and 352,980 ppm ^2^H DLW. Therefore, if they injected 300g DLW, the isotope at the equilibration time should have been around 2,900 ppm for ^18^O and 1,600 ppm for ^2^H.
3. That is different from table S1.1 (^2^H from 34 to 70; ^18^O from 66 to 130) and Nie et al do not explain how the difference arose.
4. If we assume that the y axis in Figure S1.1 is in natural log then 10 ppm of H is reached in about 200 hours, or about 8 days. So the amount of isotope in the samples for the wild animals would have been marginal for analysis at 8 to 10 days. That could have reduced the accuracy of the measurements.
5. Nie et al state that they used a syringe to inject DLW, but do not give further details. Was it a 300 ml or larger size syringe? Or did they use a smaller syringe and do multiple injections? It is important to be accurate and precise in conducting and describing methods. Is this a case of leaving out details of the methods or of a lack of care in the experiment?
6. The volume of injected fluid (≈ 300 ml) was large for an animal the size of a giant panda, and may have affected DLW estimates as well as having adverse physiological effects. There are about 3 liters of plasma and 2 liters of blood cells in a 100 kg panda. That is a total of 5 liters. The concentration of that fluid is about 300 mOsmols/L. Injecting 300 ml of water would reduce that value to about 273 mOsmols in the plasma and affect the fluid balance. The large quantity of water could lyse (break up) cells as it enters the blood system. That would cause additional physiological problems. In addition, injecting such a large amount of water would induce urination, which might cause a wash out of isotope and affect the final concentration of isotope left in the blood and body fluids. It is not possible to assess those effects based on the limited information provided by Nie et al in their article and supplementary information
7. Nie et al do not mention how they captured their wild giant pandas initially and how they recaptured them for observation and resampling after they injected DLW. They report that the giant pandas were “lightly anesthetized,” but do not clarify what that means. It is possible that the capture and handling affected the behavior and activity level of the animals for several days. Previous studies have reported that it can take 3 -5 days for free-ranging bears of other species to fully recover their activity and movement rates after aesthesia and handling^1^.

**Reference**

1. Rode, K. D, et al. Effects of capturing and collaring on polar bears: findings from long-term research on the southern Beaufort Sea population. *Wildl. Res*. **41**, 311–322 (2014)
